# Whole-genome Sequencing for Surveillance of Invasive Pneumococcal Diseases in Ontario, Canada: Rapid Prediction of Genotype, Antibiotic Resistance and Characterization of Emerging Serotype 22F

**DOI:** 10.3389/fmicb.2016.02099

**Published:** 2016-12-27

**Authors:** Xianding Deng, Nader Memari, Sarah Teatero, Taryn Athey, Marc Isabel, Tony Mazzulli, Nahuel Fittipaldi, Jonathan B. Gubbay

**Affiliations:** ^1^Public Health Ontario LaboratoryToronto, ON, Canada; ^2^Department of Laboratory Medicine and Pathobiology, University of TorontoToronto, ON, Canada; ^3^Mount Sinai Hospital and University Health NetworkToronto, ON, Canada; ^4^Department of Mathematics and Statistics, University of LavalQuebec, QC, Canada; ^5^The Hospital for Sick ChildrenToronto, ON, Canada

**Keywords:** invasive pneumococcal disease, whole-genome sequencing, emerging 22F, antibiotic resistance prediction, genomic diversification

## Abstract

**Background:** Molecular typing is essential for inferring genetic relatedness between bacterial pathogens. In this study, we applied whole genome sequencing (WGS) for rapid prediction of sequence type and antibiotic resistance for invasive pneumococcal isolates.

**Methods:** 240 isolates from adults (≥50 years old) in Ontario, Canada during 2009 to 2013 were subjected to WGS. Sequence type, antibiotic susceptibility and resistance were predicted directly from short reads. Emerging non-vaccine serotype 22F was further characterized by WGS.

**Results:** Sequence type was successfully determined for 98.3% of isolates. The overall sensitivity and specificity for antibiotic resistance prediction were 95 and 100% respectively, compared to standard susceptibility testing methods. WGS-based phylogeny divided emerging 22F (ST433) strains into two distinct clades: clade A harboring a 23 kb-prophage and anti-phage PhD/Doc system and clade B with virulence-related proteases. Five isolates in clade A developed macrolide resistance via 5.1 kb mega element recombination (encoding *mefE* and *msrD*), while one isolate in clade B displayed quinolone resistance via a *gyrA* mutation.

**Conclusions:** WGS is valuable for routine surveillance of pneumococcal clinical isolates and facilitates prediction of genotype and antibiotic resistance. The emergence of 22F in Ontario in the post-vaccine era and evidence of evolution and divergence of the 22F population warrants heightened pneumococcal molecular surveillance.

## Introduction

Invasive pneumococcal diseases (IPD) such as septicaemia and meningitis caused by *Streptococcus pneumoniae* result in a heavy burden of disease among children and adults worldwide (Ausina et al., 1988; O'Brien et al., 2007). After widespread implementation in 2002 of the 7-valent pneumococcal conjugate vaccine (PCV7, covering serotypes 4, 6B, 9V, 14, 18C, 19F, and 23F) in Canadian children, the incidence of IPD due to PCV7 serotypes decreased significantly in all age groups within the Canadian population (Kellner et al., 2009). In 2010 the new PCV13 vaccine (PCV7 serotypes plus 1, 3, 5, 6A, 7F, and 19A) replaced the PCV7 vaccine for routine immunization of children (Desai et al., 2010). Shortly after implementation of PCV13 in Canada, the number of IPD cases due to vaccine-associated serotypes such as 19A and 7F declined (Deng et al., 2015). However, the concurrent increase of IPD due to non-vaccine serotypes 22F, 15A, 33F, and 35B post-introduction of PCV13 has become a concern to clinicians (Deng et al., 2015; Golden et al., 2015; Duvvuri et al., 2016). In addition to use in children, the PCV13 vaccine was also approved by Canadian health authorities in 2012 for use among adults 50 years and older for the prevention of IPD (An Advisory Committee Statement (ACS), 2013). Currently it is also recommended for immunocompromised adults aged ≥ 18 years with high risk of IPD (An Advisory Committee Statement (ACS), 2013; Tomczyk et al., 2014).

*Streptococcus pneumoniae* is naturally competent, so it can uptake DNA from environment and undergo horizontal gene transfer between strains. Some recombination events such as deletions, insertions and inversions are frequently observed in the pneumococcal genome. In nature, horizontal gene transfer facilitates bacterial evolution, providing *S. pneumoniae* with easy access to crucial resistance genes and/or virulence factors. A recent analysis of the whole genome sequences of 240 Pneumococcal Molecular Epidemiological Network (PMEN1) pneumococcal strains revealed an exceptionally high recombination/mutation ratio of 7.2, and an average of 72 single nucleotide polymorphisms (SNPs) per recombination site (Croucher et al., 2011).

Molecular typing of *Streptococcus pneumoniae* is important for delineating genetic structure of bacterial populations and useful for inferring evolutionary relationships between isolates. For years, pulse-field gel electrophoresis (PFGE) that is based on motility variations of restriction fragments was considered the “gold standard” for subtyping of bacterial strains (Tenover et al., 1995). However, this method is technically demanding and time-consuming, and is thus not widely used for epidemiological surveillance. Multi-locus sequence typing (MLST) that measures DNA sequence variations for multiple housekeeping genes gradually replaced PFGE as a popular typing tool in the 21st century (Maiden et al., 1998). However, MLST has its shortcomings, as it is time-consuming, labor intensive and has higher cost than PFGE (Sabat et al., 2013). Recently, next-generation whole genome sequencing (WGS) has become a powerful and attractive tool for rapid typing of bacterial isolates, such as for *E. coli* (Joensen et al., 2014) and group A *streptococcus* (Athey et al., 2014). In addition, WGS has been evaluated for rapid prediction of drug susceptibility and resistance in *Mycobacterium tuberculosis* (Walker et al., 2015) and *Staphylococcus aureus* isolates (Gordon et al., 2014). With the cost of WGS continuing to decline (to a price close to or lower than MLST), it has the potential of replacing currently used typing methods for clinical isolates in the near future.

To date, MLST remains the primary method for large-scale typing of S. pneumoniae in the clinical laboratory. In this surveillance study, we employed high-throughput whole-genome sequencing for rapid prediction of MLST and antibiotic resistance *in silico* and further characterization of emerging non-vaccine serotypes of *S. pneumoniae* following the introduction of PCV13 in Ontario, Canada.

## Materials and methods

### Clinical pneumococcal isolates and antimicrobial susceptibility testing

Invasive *S. pneumoniae* isolates (1956 in total, approximately 400 isolates per year) were collected from older adults (≥50 years of age) in Ontario, Canada between 2009 and 2013. Submission of IPD isolates to Public Health Ontario Laboratories (PHOL) for further evaluation is encouraged but not mandatory. Geographic mapping of pneumococcal disease cases in Ontario older adults during 2009–2013 was done with geographic information system software QGIS (Version 2.8, http://www.qgis.org/). First 3 digits of postcode of each patient's residential address were used for the geographic mapping. Clinical isolates were recovered from sterile sites (including blood, cerebrospinal fluid and pleural fluid) and sent to the National Microbiology Laboratory (NML) in Winnipeg, Canada for serotyping using the Quellung reaction. Broth microdilution panels (Thermo Scientific, Waltham, MA, USA) were used for antimicrobial susceptibility testing for penicillin, erythromycin, clindamycin, fluoroquinolones, tetracycline, and cephalosporins. Minimum inhibitory concentrations (MICs) for each antibiotic were determined according to 2015 Clinical and Laboratory Standards Institute (CLSI) guidelines.

### Strain selection for whole genome sequencing

We randomly selected 48 strains per year from 2009 to 2013 (240 isolates in total) from all available isolates in our *S. pneumoniae* database. The number of isolates selected per serotype was proportional to all isolates in that year, with the exclusion of rare serotypes that had a frequency <1.5%. Illumina multiplex sequencing strategy was used for sequencing the 240 isolates using high-throughput processing. Genomic DNA of clinical isolates was extracted using QIAamp DNA mini kits (Qiagen, Germany). A library of multiplexed samples (96 indexes for Hiseq and 12-24 indexes for Miseq) was prepared according to standard protocols using the Nextera XT kit. Genomic libraries of 192 samples were sequenced using Illumina Hiseq 2500 (100 bp paired end, Illumina, San Diego, USA), 44 samples were sequenced using Illumina Miseq (150 bp paired end), and 4 samples were not sequenced due to poor quality of library.

### Prediction of MLST type and antibiotic resistance from short reads

The MLST sequence type was determined from short reads for each isolate using SRST2 software (Inouye et al., 2012). Genetic determinants for resistance to antibiotics including chloramphenicol, macrolides, clindamycin and tetracycline were deduced from short reads using SRST2 software by comparison with a database of resistance genes (https://github.com/katholt/srst2). The predicted susceptibilities by whole-genome sequencing were compared with phenotypic drug susceptibility test results. When there was a mismatch between phenotype and genomic prediction, phenotypic drug susceptibility tests were repeated for those isolates. Two types of errors were defined for the susceptibility portion of this study: a very major error (VME) referred to a susceptible genotype with a resistant phenotype, and a major error (ME) was defined as a resistant genotype with a susceptible phenotype.

### Genomic characterization of emerging non-vaccine serotype 22F

Genomes for 22F isolates were assembled *de novo* using Velvet 1.2.07 (Zerbino and Birney, 2008) and VelvetOptimiser (mean coverage 208x). The draft genomes had an average of 333 contigs and mean N50 of 61885 bp. The draft genomes were annotated automatically with the RAST pipeline (rast.nmpdr.org). The draft genomes of 22F have been deposited at DDBJ/EMBL/GenBank under accession MLG(H-Z)00000000 and MLH(A-F)00000000. Raw Illumina sequences of 236 isolates have been deposited at SRA/NCBI under accession SRR3211687-729, SRR5011653-846. Core genomes and accessory genomes of 22F were predicted using Pan-Genomes Analysis Pipeline (PGAP) (Zhao et al., 2012) that utilizes BLAST command line tools (NCBI), InParanoid (O'Brien et al., 2005) and MultiParanoid (Alexeyenko et al., 2006) software. The core genes shared by all 22F isolates were retrieved and concatenated to make a “core” genome. MEGA5 (Tamura et al., 2011) was used to align these core genome sequences and to visualize single nucleotide polymorphism (SNP) variants. The genome sequence alignments were imported into BratNextGen (Marttinen et al., 2012) to infer regions of recombination and for clustering analysis (grouping strains into subclusters). Gaps and SNPs due to recombination were removed. The core genome alignment file of 22F isolates was uploaded to CIPRES server (www.phylo.org) for phylogenetic analysis. A phylogenetic tree was constructed with RAxML (Stamatakis, 2014) using a GTR model with a gamma correction for site rate variation. The online tool, Interactive Tree of Life (Letunic and Bork, 2011) was used for manipulation of phylogenetic trees. For comparative genomic analysis, the accessory genomes of different groups (e.g., drug-resistant and susceptible isolates) of 22F were compared in order to identify genetic features unique to any identified groups. *Z*-test was used to predict significance of difference between the proportions of strains in the two groups possessing a gene of interest. *P* < 0.01 was considered statistically significant.

### Statistical tests

Chi-square test was used to determine the statistical difference between the proportions of emerging serotypes (i.e., 22F) relative to total isolates in each year between 2009 and 2013 (5 proportions compared using 2 × 5 contingency table). *P* < 0.05 was considered significant and a simple *Z*-test was used to compare a pair of proportions (*P* < 0.05 is considered significant).

## Results

### Emergence of non-PCV13 serotypes post-vaccination

**Figure 1:**
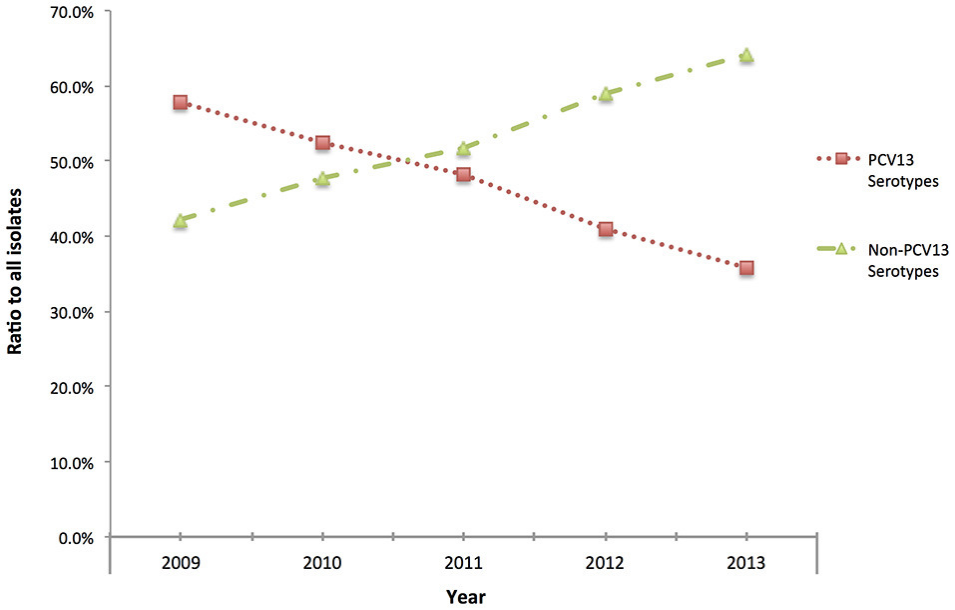
**Proportion of *Streptococcus pneumoniae* isolates causing IPD among older adults (≥50 years of age) expressing PCV13 (1, 3, 4, 5, 6A, 6B, 7F, 9V, 14, 18C, 19A, 19F, and 23F) and non-PCV13 serotypes in Ontario, Canada, during 2009–2013**.

**Figure 2:**
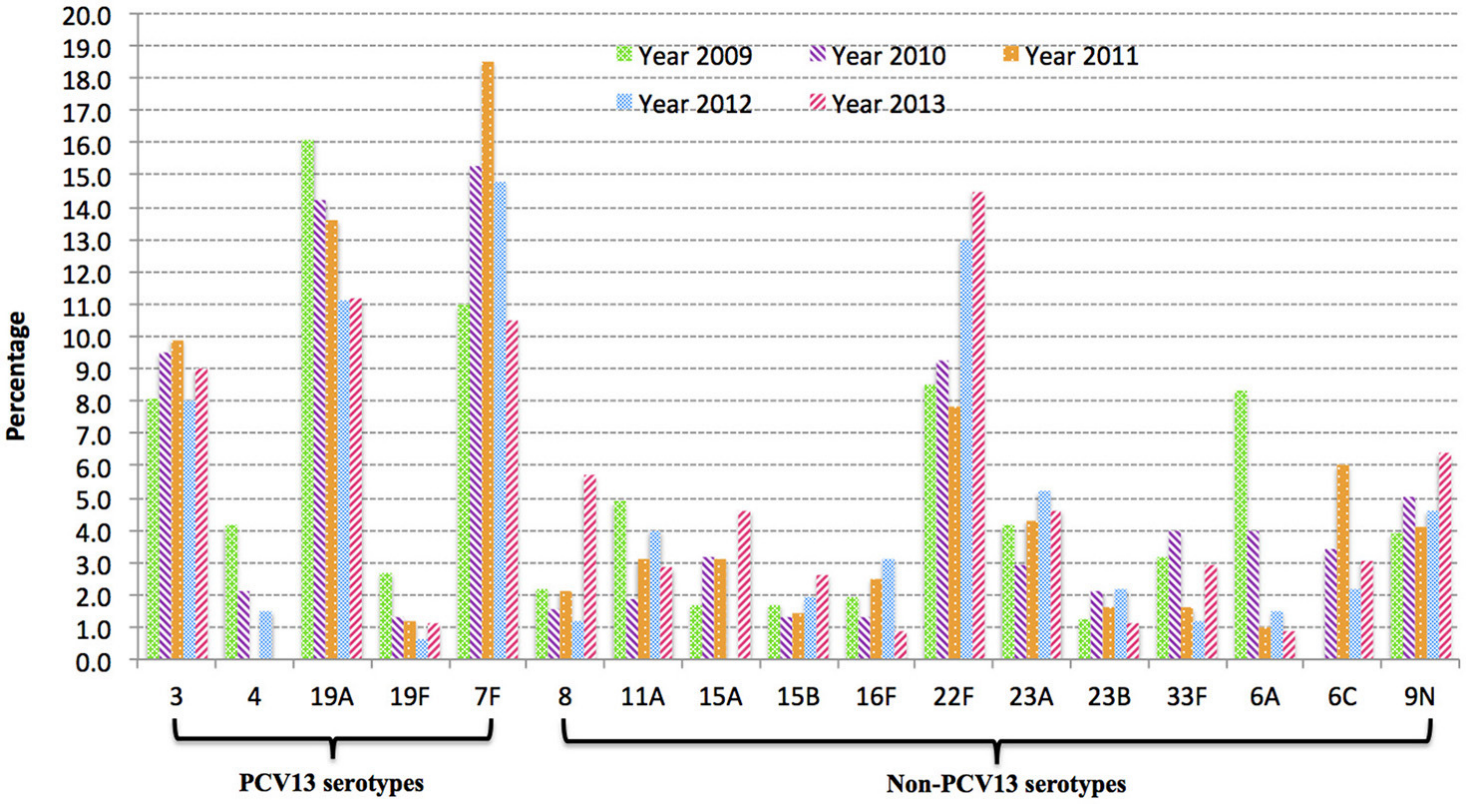
**Serotype changes over time in IPD among older adults (≥50 years of age) in Ontario, Canada from 2009 to 2013**. Serotype 22F increased significantly after introduction of PCV13 (*p* < 0.01) between 2009 and 2013, and became most prevalent in 2013.

Although, IPD isolates were voluntarily submitted to PHOL from regional health units, distribution map of clinical samples indicated a good representation of geographic regions in the province of Ontario (Figure S1). Overall, during 2009–2013 IPD cases in older adults (≥50 years of age) due to PCV13 serotypes showed the trend of decline (chi-square test *p* < 0.01 between five ratios), while IPDs due to non-PCV13 serotypes had the trend of increase (chi-square test *p* < 0.01) (Figure 1). Non-PCV13 serotype 22F increased from 8.5% in 2009 to 14.5% in 2013 (*p* < 0.01 for chi-square test, and *p* = 0.0061 bewteen 2009 and 2013) (Figure 2). In 2013, serotype 22F surpassed 19A and 7F, and became the most common *S. pneumoniae* serotype identified among IPD isolates from Ontario adults.

### Sequence type prediction *in silico* and clonal lineage analysis

WGS was performed on a total of 236 IPD isolates (48 isolates per year, with four strains excluded due to extremely low concentrations of DNA following library preparation). MLST sequence type was successfully predicted from Illumina short reads directly for 232 strains, giving a success rate of 98.3% (232/236). The four isolates that failed MLST prediction had low sequence coverage (less than 10, ranging from 2 to 8). A total of 90 different sequence types (ST) were identified and these STs could be further divided into 17 clonal groups and 39 singletons (individual STs) through eBURST analysis. The emerging post-PCV13 serotype 22F was exclusively associated with clonal complex CC433 that include ST433 (25/26, 96%) and ST4533 (1/26, 4%) a single allele variant of ST433. CC63, CC66 and CC100 were the major (>80%) CCs identified among 15A, 9N, and 33F, respectively (Table 1).

**Table 1:** **Sequence type composition of most common pneumococcal serotypes causing IPD among older patients (≥50 years old) in Ontario, Canada during 2009–2013**.

**Serotype**	**Sequence type composition**
22F	CC433 (96%, ST433, 4%, SLV^*^/4553)
19A	CC199 (36%, ST199, SLV/677/3976/2344), CC695 (36%, ST695, SLV/8323), CC416 (12%, ST416, SLV/2343), ST320 (4%), ST63 (4%), ST1201 (4%), ST5034 (4%)
7F	CC191 (93%, ST191, 7%, SLV/9363/novel ST)
3	CC180 (95%, ST180, SLV/8561/1380), ST1377 (5%)
9N	CC66 (92%, ST66, SLV/517/632/novel ST), ST4666 (8%)
8	CC404 (60%, ST404, SLV/1480, DLV/1268/novel ST), ST53 (40%)
15A	CC63 (90%, ST63, DLV/9355), ST3811 (10%)
23A	CC42 (73%, ST42, SLV/190/4726/novel ST, DLV/1839/4550), ST338 (27%)
33F	CC100 (86%, ST100, SLV/2705), ST1012 (14%)

* *SLV single-locus variant; DLV double-locus variants*.

### *In silico* antibiotic resistance prediction and comparison with phenotypic resistance testing

The presence of acquired genes (Table S1 in supplementary material) including *cat, mefA, msrD, ermB*, and *tetM* that are associated with phenotypic resistance was predicted directly from short reads for the IPD isolates sequenced. 226 (95.8%) isolates had complete concordance between genotype and phenotype for the antimicrobial agents tested. The remaining 10 isolates had a total of 11 discrepancies between genotype and phenotype (Table S2 in supplementary material). Repeated susceptibility testing for the isolates with genotype/phenotype mismatches reduced the number of discrepancies from 11 to 7, giving a revised concordance rate of 97%. There were five very major errors (0.55% VME rate) and two major errors (0.22% ME rate) for antibiotic resistance prediction of *S. pneumoniae* isolates. The overall sensitivity and specificity for predicting phenotypic resistance to the antimicrobials tested using genomic data were 95% (95% CI, 90 to 97%), and 100% (95% CI, 99 to 100%), respectively (Table 2).

**Table 2:** **Sensitivity and specificity of *in silico* genomic prediction of antibiotic resistance from short reads for *S. pneumoniae* isolates**.

**Antibiotics**	**Isolates susceptible (S) by phenotype**		**Isolates resistant (R) by phenotype**		**Total isolates**	**VME rate (%)**	**ME rate (%)**	**Sensitivity^a^ (95% CI)**	**Specificity (95% CI)**
	**Genotype S**	**Genotype R**	**Genotype S**	**Genotype R**					
Erythromycin^*^	173	0	0	37	210				
Clindamycin	208	2	1	21	232				
Chloramphenicol	226	0	2	4	232				
Tetracycline	202	0	2	28	232				
Overall	809	2	5	90	906	0.55	0.22	0.95 (0.90–0.97)	1.00 (0.99–1.00)

* *Refers to erythromycin resistance only, without phenotype of clindamycin resistance. VME, very major error (susceptible genotype with resistant phenotype); ME, major error (resistant genotype with susceptible phenotype). R, resistant; S, sensitive*.

a *Reflects sensitivity for resistance detection*.

### Phylogenetic analysis of 22F reveals two divergent subpopulations

**Figure 3:**
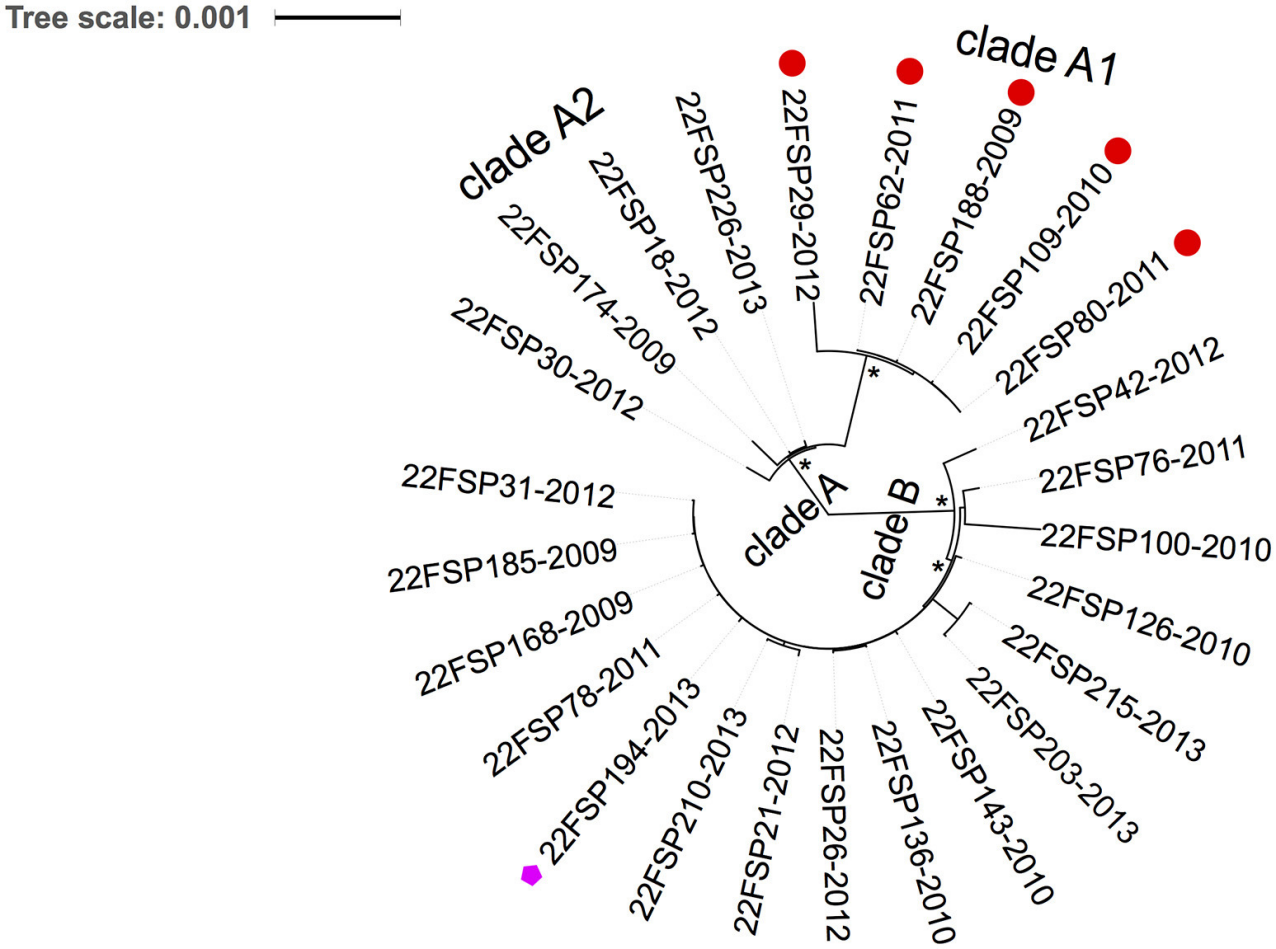
**Phylogenetic analysis of emerging 22F-ST433 isolates from Ontario, Canada between 2009 and 2013**. An un-rooted maximum-likelihood tree based on the core genome with putative recombination sites excluded. Two clades are revealed by the tree: clade A and clade B. Clade A include two sub-clades: clade A1 (five isolates) and clade A2 (four isolates). All five isolates in clade A1 were resistant to macrolides (shown with red circle at the tip), and one isolate from clade B was resistant to fluoroquinolones (pink pentagon at the tip). ^*^ indicates100% bootstrap support. Each isolate at the tip was labeled as: Serotype-isolate ID-year of isolation.

Of 26 serotype 22F isolates, 25 (96%) belonged to the same sequence type, ST433. Core genome alignment (1581 genes) of 22F-ST433 clinical isolates identified a total of 6308 SNPs. After removal of gaps and putative recombination regions predicted by BratNextgen, 3886 high-quality SNPs (2212 parsimony informative sites) were detected from 789,333 sites. A maximum likelihood phylogeny (un-rooted tree) was constructed. All 22F-ST433 isolates can be divided into two sub-clusters: clade A and clade B (Figure 3). In clade A, five isolates that confer resistance to macrolides (both erythromycin and azithromycin) are closely related to each other, forming a sub-cluster clade A1. All isolates in clade B are susceptible to macrolides and other antibiotics, except for isolate SP194, possessing resistance to levofloxacin (MIC 4 μg/mL) and moxifloxacin (MIC 2 μg/mL). Further comparison of quinolone resistance determinant regions (QRDR) between quinolone-resistant and sensitive strains found a non-synonymous mutation on *gyrA* gene (S81F) only.

### Accessory genome comparison discloses clade-specific genes in 22F

Accessory genomes of clade A and clade B isolates of 22F were compared, and presence or absence of accessory genes in each group was determined. A total of 39 genes were unique to clade A (100% presence in this group, but absent in the other), while clade B had 8 clade-specific genes (Table 3). Thirteen genes (combined length of 22,515 bp) unique to clade A were contiguous to each other (loci SP18.166056.587-610 and SP18.166056.1991-98) in the genome and encoded for a variety of bacteriophage proteins. In addition, all clade A strains possessed Type I restriction system and PhD/Doc toxin-antitoxin (TA) system in their genomes (PhD-Doc locus is surrounded by Type I restriction system and integrase). Five (55.6%) clade A isolates that form clade A1 subclade contained a gene cassette of 5163 bp (loci SP29.152236.85-92) that encode for macrolide resistance determinants *mefE* and *msrD*. The 5.1 kb gene cassette shared 96% similarity with mega (macrolide resistance genetic assembly) coding sequences upstream of *tetM* in the Tn2009 transposon previously described by Del Grosso et al. (2004). All isolates in clade B contained genes encoding for CAAX amino protease, putative UDP-glucose 6-dehydrogenase (loci SP21.166057.763-767) and several hypothetical proteins that clade A lacked.

**Table 3:** **Genome content differences between clade A and clade B of 22F clinical isolates**.

**Gene clutser ID**	**Gene locus position in draft genome**	**Gene name**	**% presence in Clade A (*n* = 9)**	**% presence in Clade B (*n* = 16)**	***P* value *Z* test**	**Comments**
2027	SP18.166056.587	Phage minor tail protein	100.00	0.00	0	Present In Clade A, absent in Clade B
2038	SP18.166056.588	Hypothetical protein	100.00	0.00	0	Present In Clade A, absent in Clade B
2006	SP18.166056.589	Phage protein	100.00	0.00	0	Present In Clade A, absent in Clade B
2000	SP18.166056.590	Hypothetical protein	100.00	0.00	0	Present In Clade A, absent in Clade B
2030	SP18.166056.591	Phage capsid and scaffold	100.00	0.00	0	Present In Clade A, absent in Clade B
2009	SP18.166056.592	Hypothetical protein	100.00	0.00	0	Present In Clade A, absent in Clade B
2005	SP18.166056.593	Hypothetical protein	100.00	0.00	0	Present In Clade A, absent in Clade B
2011	SP18.166056.594	Hypothetical protein	100.00	0.00	0	Present In Clade A, absent in Clade B
2031	SP18.166056.595	Hypothetical protein	100.00	0.00	0	Present In Clade A, absent in Clade B
2040	SP18.166056.596	Phage capsid and scaffold	100.00	0.00	0	Present In Clade A, absent in Clade B
2010	SP18.166056.597	Hypothetical protein	100.00	0.00	0	Present In Clade A, absent in Clade B
2004	SP18.166056.598	Phage minor capsid protein	100.00	0.00	0	Present In Clade A, absent in Clade B
2037	SP18.166056.599	phage portal protein	100.00	0.00	0	Present In Clade A, absent in Clade B
1998	SP18.166056.600	Phage terminase, large subunit	100.00	0.00	0	Present In Clade A, absent in Clade B
2025	SP18.166056.601	Hypothetical protein	100.00	0.00	0	Present In Clade A, absent in Clade B
2042	SP18.166056.603	Hypothetical protein	100.00	0.00	0	Present In Clade A, absent in Clade B
2034	SP18.166056.604	Hypothetical protein	100.00	0.00	0	Present In Clade A, absent in Clade B
2022	SP18.166056.605	Phage protein	100.00	0.00	0	Present In Clade A, absent in Clade B
2369	SP18.166056.606	Hypothetical protein	100.00	0.00	0	Present In Clade A, absent in Clade B
2389	SP18.166056.607	Hypothetical phage protein	100.00	0.00	0	Present In Clade A, absent in Clade B
2017	SP18.166056.608	Phage Holliday junction resolvase	100.00	0.00	0	Present In Clade A, absent in Clade B
738	SP18.166056.609	Hypothetical protein	100.00	0.00	0	Present In Clade A, absent in Clade B
2021	SP18.166056.610	Hypothetical protein	100.00	0.00	0	Present In Clade A, absent in Clade B
1921	SP18.16605.1991	lysozyme family	100.00	0.00	0	Present In Clade A, absent in Clade B
2044	SP18.166056.1992	Hypothetical protein	100.00	0.00	0	Present In Clade A, absent in Clade B
2043	SP18.166056.1994	putative prophage protein	100.00	0.00	0	Present In Clade A, absent in Clade B
2039	SP18.166056.1995	Hypothetical protein	100.00	0.00	0	Present In Clade A, absent in Clade B
2002	SP18.166056.1996	phage tail length tape measure protein	100.00	0.00	0	Present In Clade A, absent in Clade B
2024	SP18.166056.1997	Hypothetical protein	100.00	0.00	0	Present In Clade A, absent in Clade B
2003	SP18.166056.1998	Phage tail length tape-measure protein	100.00	0.00	0	Present In Clade A, absent in Clade B
2051	SP18.166056.519	Type I restriction-modification system	100.00	0.00	0	Present In Clade A, absent in Clade B
2012	SP18.166056.520	antitoxin PhD	100.00	0.00	0	Present In Clade A, absent in Clade B
1999	SP18.166056.521	death on curing protein, Doc toxin	100.00	0.00	0	Present In Clade A, absent in Clade B
2016	SP18.166056.522	phage integrase	100.00	0.00	0	Present In Clade A, absent in Clade B
2007	SP18.166056.858	replication protein	100.00	0.00	0	Present In Clade A, absent in Clade B
2013	SP18.166056.714	Hypothetical protein	100.00	0.00	0	Present In Clade A, absent in Clade B
2020	SP18.166056.1278	Phage integrase	100.00	0.00	0	Present In Clade A, absent in Clade B
2023	SP18.166056.708	Hypothetical protein	100.00	0.00	0	Present In Clade A, absent in Clade B
2033	SP18.166056.711	Hypothetical protein	100.00	0.00	0	Present In Clade A, absent in Clade B
2085	SP21.166057.763	CAAX amino protease I	0.00	100.00	0	Present In Clade B, absent in Clade A
2138	SP21.166057.764	CAAX amino protease II	0.00	100.00	0	Present In Clade B, absent in Clade A
2077	SP21.166057.765	Hypothetical protein	0.00	100.00	0	Present In Clade B, absent in Clade A
2148	SP21.166057.767	UDP-glucose 6-dehydrogenase	0.00	100.00	0	Present In Clade B, absent in Clade A
2170	SP21.166057.769	Hypothetical protein	0.00	100.00	0	Present In Clade B, absent in Clade A
2101	SP21.166057.1168	Hypothetical protein	0.00	100.00	0	Present In Clade B, absent in Clade A
2171	SP21.166057.482	Hypothetical protein	0.00	100.00	0	Present In Clade B, absent in Clade A
2172	SP21.166057.677	Hypothetical protein	0.00	100.00	0	Present In Clade B, absent in Clade A
2265	SP29.152236.85	Novel pyridoxal kinase, thiD family	55.56	0.00	0.00086	Overrepresented in Clade A, absent in Clade B
2271	SP29.152236.86	ImpB/MucB/SamB family protein	55.56	0.00	0.00086	Overrepresented in Clade A, absent in Clade B
2270	SP29.152236.87	Hypothetical protein	55.56	0.00	0.00086	Overrepresented in Clade A, absent in Clade B
2276	SP29.152236.88	Hypothetical protein	55.56	0.00	0.00086	Overrepresented in Clade A, absent in Clade B
2272	SP29.152236.89	Hypothetical protein	55.56	0.00	0.00086	Overrepresented in Clade A, absent in Clade B
1435	SP29.152236.90	marolide-resistance protein (msrD/mel)	55.56	0.00	0.00086	Overrepresented in Clade A, absent in Clade B
2283	SP29.152236.91	Macrolide-efflux protein (mefE)	55.56	0.00	0.00086	Overrepresented in Clade A, absent in Clade B
2268	SP29.152236.92	unknown protein	55.56	0.00	0.00086	Overrepresented in Clade A, absent in Clade B
2292	SP30.166058.302	Hypothetical protein	77.78	0.00	0	Overrepresented in Clade A, absent in Clade B
2291	SP30.166058.303	Hypothetical protein	77.78	0.00	0	Overrepresented in Clade A, absent in Clade B
2296	SP30.166058.304	Hypothetical protein	44.44	0.00	0.00362	Overrepresented in Clade A, absent in Clade B
2267	SP29.152236.408	Ribulose-phosphate 3-epimerase (RPE)	55.56	0.00	0.00086	Overrepresented in Clade A, absent in Clade B
1510	SP29.152236.409	PTS fructose transporter subunit IIC	55.56	0.00	0.00086	Overrepresented in Clade A, absent in Clade B
2273	SP29.152236.410	PTS fructose transporter subunit IIB	55.56	0.00	0.00086	Overrepresented in Clade A, absent in Clade B
2275	SP29.152236.411	PTS fructose transporter subunit IIA	55.56	0.00	0.00086	Overrepresented in Clade A, absent in Clade B
2280	SP29.152236.412	PTS fructose transporter subunit IIA	55.56	0.00	0.00086	Overrepresented in Clade A, absent in Clade B
2269	SP29.152236.1295	Hypothetical protein	55.56	0.00	0.00086	Overrepresented in Clade A, absent in Clade B
2266	SP29.152236.1057	Mobile element protein	44.44	0.00	0.00362	Overrepresented in Clade A, absent in Clade B
2282	SP29.152236.1087	Hypothetical protein	55.56	0.00	0.00086	Overrepresented in Clade A, absent in Clade B
2284	SP29.152236.732	Hypothetical protein	55.56	0.00	0.00086	Overrepresented in Clade A, absent in Clade B
2290	SP30.166058.1442	Hypothetical protein	77.78	0.00	0	Overrepresented in Clade A, absent in Clade B
2322	SP62.152237.1836	Hypothetical protein	44.44	0.00	0.00362	Overrepresented in Clade A, absent in Clade B
1996	SP18.166056.1712	Transposase	55.56	0.00	0.00086	Overrepresented in Clade A, absent in Clade B
2035	SP18.166056.1403	Mobile element protein	88.89	0.00	0	Overrepresented in Clade A, absent in Clade B
2038	SP18.166056.588	Hypothetical protein	88.89	0.00	0	Overrepresented in Clade A, absent in Clade B

## Discussion

In a previous study we used the Sanger-sequencing based MLST method for the molecular typing of *S. pneumoniae* isolates from Ontario children (Deng et al., 2015). However, MLST typing is time consuming and very labor intensive. For example, to type 100 pneumococcal isolates using seven gene loci, 700 PCR amplifications plus 1400 Sanger sequencing reactions (forward and reverse direction) are required. Additional repeated sequencing is often needed for ambiguous bases in a sequence due to a variety of reasons such as poor DNA quality and contaminations. Compared to MLST and PFGE, WGS has the highest discriminatory power and has great potential for large-scale typing of bacterial isolates (Joensen et al., 2014). In this surveillance study, we used WGS for rapid determination of sequence type and antibiotic resistance for IPD isolates submitted to a public health laboratory. Illumina high-throughput genome sequencing combined with multiplexing allows rapid sequencing of hundreds of samples in a short period of time (1–5 days in rapid run mode). Our results suggest that MLST type could be accurately predicted from Illumina short reads directly for *S. pneumoniae*, provided genome sequences have fairly good sequencing depth (at least 10 or better). The overall sensitivity and specificity of genomic prediction of antibiotic resistance for *S. pneumoniae* were 95% (95% CI, 90 to 97%), and 100% (95% CI, 99 to 100%) respectively, compared to standard susceptibility testing methods. Ninety-seven percent of samples had complete concordance between genotype and phenotype for the antimicrobial agents tested, with low rates of VME (0.55%) and ME (0.22%). These results suggest that WGS prediction of antibiotic resistance mechanisms acquired through horizontal gene transfer events was as sensitive and specific as routine antimicrobial susceptibility assays for pneumococci. However, it is worth noting that only four antibiotics were tested in this study, and our observations in pneumococci may not apply to other bacterial pathogens. In addition, this WGS method is currently unable to reliably predict some resistance mechanisms due to point mutations on gene loci. For example, penicillin resistance among pneumococcal isolates resulting from mutations in penicillin-binding proteins (PBPs) or non-PBP genes cannot be predicted. Although, current phenotypic susceptibility assays are likely to be more reliable for routine testing, WGS methods may be useful for slow growing organisms, or organisms that cannot be cultured.

We have detected some VME and ME errors when comparing WGS methods with standard phenotypic susceptibility tests. Possible explanations for VMEs (genotype-susceptible but phenotype-resistant) may be sequencing error, alternative resistance mechanisms that are not reflected in the resistance gene database and novel resistance mechanisms not previously described. MEs (phenotypically susceptible isolates that contain resistance genes) may be the result of suppressed or delayed gene expression of resistance genes, since expression of a gene is a complex process that involves the interactions between repressors, promoters and other regulatory networks.

In our previous study we reported the increase of 22F prevalence among children (≤ 5 years of age) in Ontario in the post-PCV13 era (Deng et al., 2015). In the present study a significant increase in 22F-associated IPD cases was observed in older adults in Ontario (≥ 50 years old) after the introduction of PCV13. In 2013, 22F was the most common serotype detected in IPD among all ages in Ontario, Canada. The emergence of 22F in the post-vaccine era was also reported in other regions of Canada (Demczuk et al., 2013), and in other countries such as the USA (Kendall et al., 2016), UK (Pichon et al., 2013), Germany (van der et al., 2015), and Norway (Steens et al., 2015). An extensive review of serotype 22F in a MLST database found that it was broadly distributed in over 25 countries and the earliest report of IPD caused by 22F was in 1993 (MLST Databases, 2016). According to the public MLST database, 22F capsular type is mainly associated with the MLST-defined clonal complex CC433 (over 70%), ST1294 (3.3%) and CC698 (3.1%) (MLST Databases, 2016). Although, 24/25 (96%) Ontario 22F isolates mostly shared identical genotype (ST433), WGS-based phylogenetic analysis divided the 22F population into two distinct clusters (clade A and B in Figure 2) that were diverse in their genomic contents. The discovery of clade-specific gene sets by comparative genomics suggests the diversification of 22F-ST433 clone. Interestingly, all strains in clade A shared a 23kb-prophage-associated genomic island that clade B lacked. This chromosomal island comprises 30 genes including lysozyme genes downstream from the phage minor tail protein gene. To combat the bacteriophage invasion, clade A strains possessed anti-phage weaponry of Type-I restriction system that degrades phage DNA (Loenen et al., 2014) and PhD/Doc TA system which promotes cell death and limits phage replication within bacterial populations (Fineran et al., 2009). In addition, clade A1 (5 isolates) acquired macrolide resistance through incorporation of a 5.1 kb mega gene cassette that encodes for *mefE* and *msrD* resistance genes possibly via horizontal gene transfer. Antibiotic resistance obtained by 22F might provide selective advantage for them over other drug-susceptible competitors. In the current study 19.2% of 22F isolates carried this macrolide-resistant gene cassette; further dissemination of drug resistance among 22F should be closely monitored. 22F isolates in clade B possessed CAAX proteases that may be virulence-related (Firon et al., 2013) and their exact functions are unknown. One strain in clade B, isolated in 2013, demonstrated fluoroquinolone resistance through a *de novo gyrA* gene mutation (S81F). These gene differences between distinct clades suggest ongoing evolution and divergence of 22F clone through genetic recombination possibly due to rapid adaptation to environmental stress and selection pressure.

In conclusion, our study suggests that WGS is a valuable tool for routine molecular typing of pneumococcal isolates in the clinical laboratory, including antibiotic resistance prediction. The emergence of 22F in Ontario, Canada and its population divergence warrants heightened surveillance. Before routine use of WGS within clinical laboratories, certain barriers need to be addressed such as sequencing quality assurance, the need for automated WGS data analysis tools, and a shortage of qualified personnel with the necessary skills for data interpretation.

## Author contributions

NM prepared DNA library for genome sequencing. XD performed data analysis and prepared the manuscript. ST and TA performed bioinformatic analysis. MI performed geographic mapping of isolates. TM helped data analysis and manuscript writing. NF and JG designed and supervised this project.

## Funding

This study was supported by Pfizer, Canada and Public Health Ontario.

### Conflict of interest statement

JG has received research grants from GlaxoSmithKline Inc., and Hoffman-La Roche Ltd., to study antiviral resistance in influenza, and from Pfizer Inc., to conduct surveillance of *Streptococcus pneumoniae*. TM has served on advisory boards for Pfizer Inc., and Merck Inc. All other authors declare that the research was conducted in the absence of any commercial or financial relationships that could be construed as a potential conflict of interest.

## Supplementary material

Figure S1 **Geographic distribution of invasive pneumonia disease cases among Ontario older adults (≥50 years of age) between 2009 and 2013**.

Table S1 **List of acquired genes predicted *in silico* from genome sequences that correlates with phenotype resistance**.

Table S2 **Details of antibiotic resistance discrepancies between phenotype test and genotype prediction**.
