# Supplementary figures and images for: Whole-genome Sequencing for Surveillance of Invasive Pneumococcal Diseases in Ontario, Canada: Rapid Prediction of Genotype, Antibiotic Resistance and Characterization of Emerging Serotype 22F
Source: Front Microbiol. 2016 Dec 27;7:2099. doi: 10.3389/fmicb.2016.02099 (PMC5187366; doi:10.3389/fmicb.2016.02099)

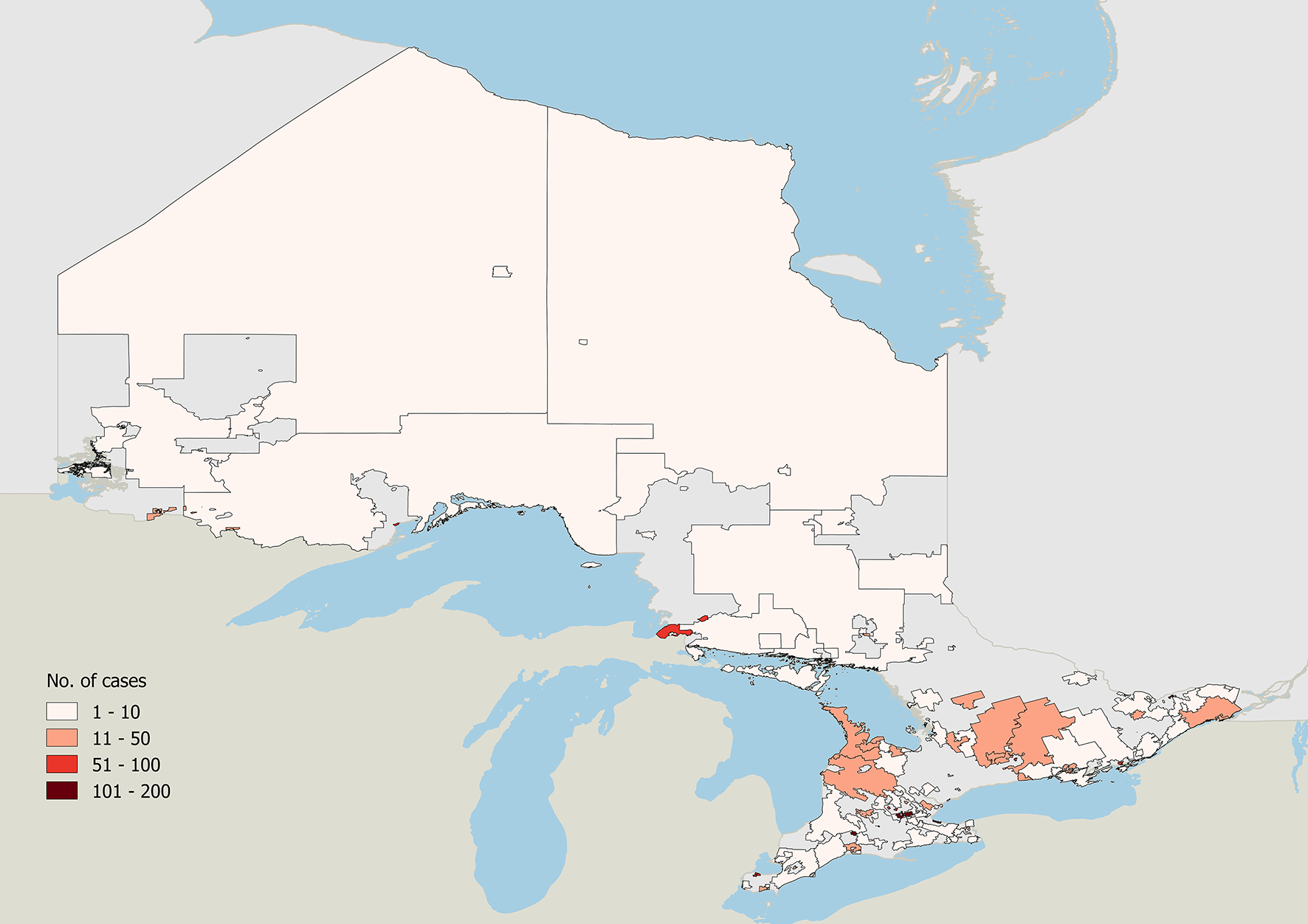

Supplement: Figure S1 — Geographic distribution of invasive pneumonia disease cases among Ontario older adults (≥50 years of age) between 2009 and 2013. [file Image1.TIFF]
