# Supplementary material for: Whole-genome Sequencing for Surveillance of Invasive Pneumococcal Diseases in Ontario, Canada: Rapid Prediction of Genotype, Antibiotic Resistance and Characterization of Emerging Serotype 22F
Source: Front Microbiol. 2016 Dec 27;7:2099. doi: 10.3389/fmicb.2016.02099 (PMC5187366; doi:10.3389/fmicb.2016.02099)
Supplement: Table S1 — List of acquired genes predicted in silico from genome sequences that correlates with phenotype resistance. [file Table1.docx]

**Table S1. List of acquired genes predicted *in silico* from genome sequences that correlates with phenotype resistance**

| Antimicrobial agents | Gene | Coding protein | Reference gene accession number |
| --- | --- | --- | --- |
| Erythromycin | *mefA* | macrolide resistant protein | AF376746 |
| Erythromycin | *msrD* | macrolide resistant protein | AF274302 |
| Erythromycin/ clindamycin | *ermB* | rRNA adenine N-6-methyltransferase | JN899585 |
| Tetracycline | *tetM* | ribosomal protection protein | X04388 |
| Chloramphenicol | *cat* | chloramphenicol acetyltransferase | NC_002013 |
