# Supplementary material for: Whole-genome Sequencing for Surveillance of Invasive Pneumococcal Diseases in Ontario, Canada: Rapid Prediction of Genotype, Antibiotic Resistance and Characterization of Emerging Serotype 22F
Source: Front Microbiol. 2016 Dec 27;7:2099. doi: 10.3389/fmicb.2016.02099 (PMC5187366; doi:10.3389/fmicb.2016.02099)
Supplement: Table S2 — Details of antibiotic resistance discrepancies between phenotype test and genotype prediction. [file Table2.docx]

Table S2. Details of antibiotic resistance discrepancies between phenotype test and genotype prediction

| Type of errors and antibiotics | Isolate number | Initial susceptibility phenotype | Phenotype after repeat testing | Genotype results |
| --- | --- | --- | --- | --- |
| VME* |  | | | |
| Erythromycin | SP91 | R | S | No *mef* or *msrD* gene detected |
| Erythromycin/  clindamycin | SP81 | R/R | R/R | No *ermB* gene detected |
| Chloram-phenicol | SP171  SP197  SP212 | R  R  R | S  R  R | No *cat* gene detected  No *cat* gene detected  No *cat* gene detected |
| Tetracycline | SP99  SP157  SP221 | R  R  R | S  R  R | No *tetM* gene detected  No *tetM* gene detected  No *tetM* gene detected |
| ME** |  | | | |
| Erythromycin/  clindamycin | SP50  SP182 | S  S | S  S | *ermB* gene detected  *ermB* gene detected |
| Chloram-phenicol | SP182 | S | R | *cat(pC194)* gene detected |

*VME, very major error (susceptible genotype with resistant phenotype); **ME, major error (resistant genotype with susceptible phenotype). R, resistant; S, sensitive.
